# Interaction of a CD44+ head and neck squamous cell carcinoma cell line with a stromal cell-derived factor-1-expressing supportive niche: An *in vitro* model

**DOI:** 10.3892/ol.2013.1673

**Published:** 2013-11-11

**Authors:** ANNE FABER, CHRISTOPH ADERHOLD, ULRICH REINHART GOESSLER, KARL HOERMANN, JOHANNES DAVID SCHULTZ, CLAUDIA UMBREIT, UTE WALLICZEK, JENS STERN-STRAETER

**Affiliations:** Department of Otorhinolaryngology, Head and Neck Surgery, University Medical Centre Mannheim, Mannheim D-68167, Germany

**Keywords:** head and neck squamous cell carcinoma, cancer stem cells, stem cell niche, SDF-1, CXCR4

## Abstract

The cancer stem cell (CSC) theory implies that CSCs are surrounded by supportive stromal cells, which are known as the CSC niche. Stromal cell-derived factor-1 (SDF-1) shows a multitude of functional effects in head and neck squamous cell carcinoma (HNSCC) cells, including migration and polarization. Therefore, the SDF-1-CXCR4 axis may be involved in the pathophysiology of the progression, recurrence and metastasis of malignant diseases of the head and neck. In the present study, the CD44^+^ HNSCC UM-SCC-11A cell line was used as a model for CSCs. The interaction between the UM-SCC-11A cells and the supportive microenvironmental cells, including fibrocytes, human umbilical vein endothelial cells (HUVECs) and human microvascular vein endothelial cells (HMVECs) was evaluated. All the cell types that were tested were shown to secrete different concentrations of SDF-1 into the surrounding culture medium [mean (m)_fibro_, 1243.3±156.2 pg/ml; m_HMVEC_, 1061.4±23.2 pg/ml; m_HUVEC_, 849.6±110.9 pg/ml]. The migration of the UM-SCC-11A cells towards the supportive cells was increased by a higher supply of SDF-1 (contr_fibro_, 315.23±61.55 μm; m_fibro_, 477.73±143.7 μm; P_fibro_=0.003; contr_HMVEC_, 123.41±66.68 μm; m_HMVEC_, 249.04±111.95 μm; P_HMVEC_=0.004; contr_HUVEC_, 189.7±93.26 μm; m_HUVEC_, 260.82±161.58 μm). The amount of the UM-SCC-11A cells that migrated towards the differentiated fibrocytes was significantly higher than that which migrated towards the HMVECs or HUVECs (P_fibro/HMVEC_=2.12E-11; P_fibro/HUVEC_=2.28E-5). Cell-cell interaction by podia formation of the UM-SCC-11A cells was observed in all the supportive cell types that were tested. Broadly based cell-cell contacts were observed. By contrast, digitiform podia formations presented by the UM-SCC-11A cells were determined using fluorescence microscopy. The SDF-1-CXCR4 axis is postulated to be a crucial pathway in the interaction between CSCs and their surrounding supportive cells. Understanding the cell-cell interactions in the CSC niche using *in vitro* models may aid in gaining further insight into these mechanisms and finding new strategies of therapy in this field.

## Introduction

Although advances have been made in the surgical and conservative therapy of head and neck squamous cell carcinoma (HNSCC), the mortality rate from this disease has remained stable over the last years (1). This is mainly due to the development of therapy-resistant local and regional recurrences (2). Antineoplastic treatments, including chemotherapy or radiation, may efficiently eradicate the majority of proliferating cells within malignant tumors. However, there is increasing evidence that there is a subpopulation of resistant tumor cells that are resistant to these regimens. These cancer stem cells (CSCs) have distinct features of somatic stem cells, including self-renewal, proliferation and differentiation. Therefore, these cells are essential and responsible for the initiation, but also the maintenance and recurrence, of malignant disease (3). The CSC hypothesis has previously been applied to HNSCC (4–6). Prince *et al* revealed that CD44^+^ cancer cells, which typically comprise <10% of the cells in a HNSCC tumor, but not CD44^−^ cancer cells, gave rise to new tumors *in vivo*(4). CD44^+^ cells in tumors of the head and neck are therefore referred to as the CSCs of HNSCC.

Stromal cell-derived factor-1 (SDF-1), also known as CXCL12, has variable effects on a plurality of cells (7). CXCR4 has been identified as its corresponding receptor. The SDF-1-CXCR4 axis is postulated to be a key pathway in the interaction between CSCs and the surrounding supportive cells in the CSC niche, and this has mainly been shown in the hematopoietic system (7,8).

SDF-1 is a multifunctional cytokine that is expressed and secreted by several tissues, including endothelial and stromal cells (9,10), which are one component of the bulk of a HNSCC tumor. There is increasing evidence that the tumor stroma also plays a significant role in terms of the response to therapeutic interventions, including chemotherapy (11). SDF-1 has a single open reading frame of 282 nucleotides that encodes a polypeptide of 93 amino acids. The cytokine arises in two isoforms, SDF-1α (24–88 amino acids) and SDF-1β (24–93 amino acids) by alternative splicing (10,12,13). SDF-1 has been shown to be a potent chemoattractant for hematopoietic progenitor cells (HPCs) and induces directional locomotion and podia formation in HNSCC in a dose-dependent manner (14). Therefore, SDF-1 is considered to be one of the key regulators for HPC trafficking between the peripheral blood circulation, bone marrow (9,10,15,16) and in the CSC niche of HNSCC.

The analysis of the CSC niche theory, where CSCs are in contact with their surrounding supportive cells, may provide information regarding cell trafficking and the underlying mechanisms of cancer, including tumor expansion, recurrence and metastatic progress. The interaction between SDF-1 and its receptor, CXCR4, may play a significant role in the CSC niche of HNSCC and other malignant epithelial tumors.

The present study monitored the interaction between the CD44^+^ UM-SCC-11A cell line and potentially supportive microenvironmental cells as an *in vitro* model for the stem cell niche in HNSCC. Fibrocytes, human umbilical vein endothelial cells (HUVECs) and human microvascular vein endothelial cells (HMVECs) served as potential counterparts to CSCs in this model. The development of *in vitro* models that imitate cellular interactions in cancer is essential for the evaluation of potential therapeutic agents. The function of SDF-1 can be mimicked by small peptide agonists, for example CTCE-0214 (10). These molecules have several advantages over the natural substances, such as ease of manufacturing. It is possible that such peptide agonists of SDF-1 comprise new strategies of therapeutical intervention in HNSCC. The cancer stem cell theory requires confirmation by further experiments.

## Materials and methods

### Cell lines and cell culture

The HNSCC cell line UM-SCC-11A was obtained from Dr T E Carey (University of Michigan, Ann Arbor, MI, USA). The cell line originates from a primary human HNSCC from the larynx of a male patient who did not undergo treatment prior to the excision (17). The cell culture of the UM-SCC-11A cells was performed in Dulbecco’s modified Eagle’s medium (DMEM; Fisher Scientific Co., Pittsburgh, PA, USA) supplemented with 10% fetal calf serum (FCS) and antibiotics (Life Technologies Inc., Gaithersburg, MD, USA).

The HUVECs (Promocell, Heidelberg, Germany) were cultured in Endothelial Cell Growth Medium (C-22010; Promocell) supplemented with additives (C-39215; 0.4% endothelial cell growth supplement/heparin (ECGS/H), 2% FCS+0.1 ng/ml epidermal growth factor+1 μg/ml hydrocortisone+1 ng/ml basic fibroblast factor; Promocell). The HMVECs (Clonetics Corp., San Diego, CA, USA) were cultured in Endothelial Cell Growth Medium MV (C-22020; Promocell) with additives (C-39225; 0,4% ECGS/H+5% FCS+10 ng/ml epidermal growth factor+1 μg/ml hydrocortisone; Promocell). The fibrocytes were obtained from the skin of a patient at the Department of Otorhinolaryngology Head and Neck Surgery, University Medical Centre Mannheim (Mannheim, Germany) who was administered radiation. The fibrocytes were raised in DMEM high glucose with additives (C-71210; 10% FCS+2% 200 mM L-glutamin+1% pen/strep/Fungizone; Promocell). Written informed consent was obtained from the patient. The culture of the HUVECs, HMVECs and fibrocytes was performed in gelatine-coated culture flasks. Confluent monolayers were passaged by trypsin. The cell culture of all the cell lines was performed at 37°C in a 5% CO_2_ fully humidified atmosphere.

### Enzyme-linked immunosorbent assay (ELISA)

The transmission of SDF-1 by the cell lines was measured using a human SDF ELISA kit (R&D Systems, Wiesbaden, Germany). A monoclonal antibody against soluble SDF-1 was adsorbed to the microwells in 96-well microtiter plates. The samples, including standards of known SDF-1 concentrations and the samples that were tested, were pipetted into the wells. During the first incubation, the SDF-1 antigen was added to wells. Subsequent to being washed, a biotinylated monoclonal antibody that was specific for SDF-1 was incubated and the streptavidin-peroxidase enzyme was added. Following the incubation period and washing to remove all the unbound enzyme, a substrate solution was added, which catalyzed a reaction on the bound enzyme and induced a colored reaction product. The intensity of this colored product was directly proportional to the concentration of SDF-1 that was present in the samples.

### Immunofluorescence labeling

To detect the expression of CD44 as a CSC marker in the UM-SCC-11A line and of CD105 in the fibrocytes, HMVECs and HUVECs, the cells were incubated with CD44/CD105 antibody (mouse monoclonal; 1:100; Abcam, Cambridge, UK) in order to observe the cell membrane staining for 1 hour at 37°C, followed by incubation with a second biotinylated antibody (anti-mouse, 1:100) for 30 min. Following further washing steps with phosphate-buffered saline, the cells were treated with streptavidin-Cy3 (1:1,000)/Streptavidin-Alexia 488 (1:500) (Jackson ImmunoResearch Inc., West Grove, PA, USA) for 30 min at room temperature. The cell nuclei were stained by DAPI. Immunofluorescence labeling using CD44/CD105 was used to prepare the cells for an evaluation of cell-cell-interaction and podia formation.

### Fluorescence microscopy

The analysis of the cell morphology, cell-cell interaction and podia formation was performed as follows. The CD44^+^ UM-SCC-11A cells (with green fluorescence using Alexia 488) and CD105^+^ fibrocytes, HMVECs and HUVECs (with red fluorescence via Cy 3) were seeded in DMEM (Fisher Scientific Co.), supplemented with 10% FCS and antibiotics (Life Technologies Inc.) and incubated to promote podia formation and contact. Following this, the cell morphology was assessed using fluorescence microscopy subsequent to the cells being fixed.

### Migration assay

Chemotaxis was assessed using an *in vitro* two-chamber transwell assay. The fibrocytes, HUVECs or HMVECs were added to the lower section of the transwell chamber (8.0 μm pore size, 6.5 mm diameter inserts; Costar Inc., Union City, CA, USA). Equal cell numbers of UM-SCC-11A were seeded in the upper chamber in medium that did not contain SDF-1. After 24 h, the transwells were removed and the cells that had migrated through the micropores were counted. UM-SCC-11A cells are adherent cells. When migrated through pores of the upper well of the migration assay, they do not drop to the bottom of the lower well. They remain adherent to the bottom of the upper well, which makes it difficult to count them. However, they form a cell-ring on the bottom that may be colored and the width may be measured as a indicator of cell count. A total of four experiments were performed.

### Statistical analysis

All results are presented as the means ± SD. Student’s t-test (two-tailed distribution, two-sample equal variance) was used to estimate the probability of the differences. P<0.05 was considered to indicate a statistically significant difference.

## Results

### Expression of CD44 in the UM-SCC-11A cells

Immunofluorescence labeling of UM-SCC-11A was performed. CD44, as a stem cell marker in HNSCC, was visualized as green fluorescence by immunofluorescence labeling using Alexia 488. In the UM-SCC-11A cells, an intense green fluorescence signal of all the cells was detected by marking CD44. CD44 was mainly expressed on the cell surface in all the samples that were stained, which allowed the evaluation of the cell-cell interaction and podia formation of the UM-SCC-11A cells towards the potentially supportive cell types that were used in the experiments (fibrocytes, HUVECs and HMVECs).

### Transmission of SDF-1 by supportive cell lines

**Figure 1:**
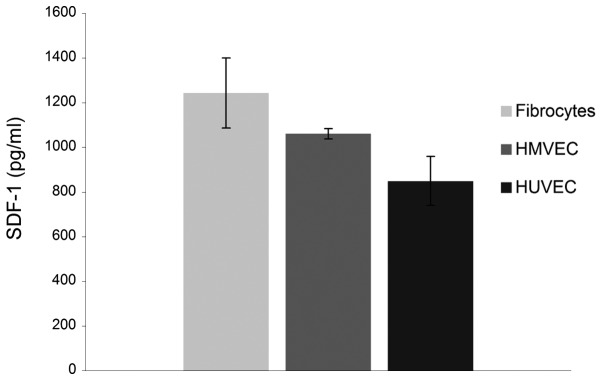
Expression of SDF-1 by the supportive cellular microenvironment in the CSC niche. ELISA analysis was performed to measure the expression of SDF-1 by fibrocytes, HMVECs and HUVECs. The values are presented as the mean ± standard deviation. m_fibro_, 1243.3±156.2 pg/ml; m_HMVEC_, 1061.4±23.2 pg/ml; m_HUVEC_, 849.6±110.9 pg/ml. SDF-1, stromal cell-derived factor-1; CSC, cancer stem cell; HMVEC, human microvascular vein endothelial cell; HUVEC, human umbilical vein endothelial cell.

An ELISA analysis was performed to measure the transmission of SDF-1 by fibrocytes, HMVECs and HUVECs. The level of SDF-1 that was secreted into the culture medium by the fibrocytes, HMVECs and HUVECs is shown in Fig. 1. The highest concentration was observed in the fibrocytes, followed by the HMVECs. The lowest concentration of SDF-1 was observed in the HUVECs. The values were determined as m_fibro_, 1243.3±156.2 pg/ml and m_HMVEC_, 1061.4±23.2 pg/ml; m_HUVEC_, 849.6±110.9 pg/ml (Fig. 1).

### Podia formation of UM-SCC-11A cells and cell-cell interaction with supportive cell lines

Polarization and podia formation are prerequisites for the directional locomotion of cells. The present study analyzed podia formation and cell-cell interaction between CD44^+^ UM-SCC-11A cells and CD105^+^ fibrocytes, HMVECs and HUVECs as a model for the CSC niche of HNSCC. For this purpose, fluorescence labeling of the UM-SCC-11A cells (CD44^−^ Alexia 488, green fluorescence) and of the fibrocytes, HMVECs and HUVECs (CD105^−^ Cy 3, red fluorescence) was performed. Direct cell-cell interaction was observed in terms of podia formation and adhesion of the UM-SCC-11A cells to the supportive stromal cell types. The cell-cell interactions were observed as broadly based cell contacts (Fig. 3A). However, digitiform podia formations of the UM-SCC-11A cells towards the supportive stromal cells were also identified (Fig. 3B).

### Migration of UM-SCC-11A cells towards supportive cell lines

**Figure 2:**
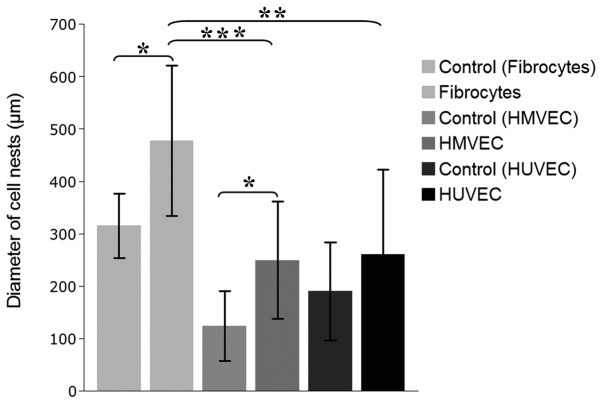
Migration of UM-SCC-11A cells towards the supportive microenvironmental stromal cells. The migration of the CD44^+^ UM-SCC-11A towards the supportive stromal cells was increased by a higher supply of SDF-1. The values are presented as the mean ± standard deviation. contr_fibro_, 315.23±61.55 μm; m_fibro_, 477.73±143.7 μm; ^*^P_fibro_=0.003; contr_HMVEC_, 123.41±66.68 μm; m_HMVEC_, 249.04±111.95 μm; ^*^P_HMVEC_=0.004; contr_HUVEC_, 189.7±93.26 μm; m_HUVEC_, 260.82±161.58 μm. The amount of UM-SCC-11A migrating was significantly higher towards the differentiated fibrocytes compared with the HMVECs or HUVECs (^***^P_fibro/HMVEC_=2.12E-11; ^**^P_fibro/HUVEC_=2.28E-5). SDF-1, stromal cell-derived factor-1; CSC, cancer stem cell; HMVEC, human microvascular vein endothelial cell; HUVEC, human umbilical vein endothelial cell.

**Figure 3:**
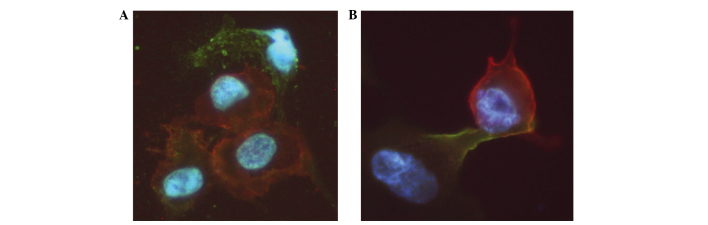
Interaction between CSCs in HNSCC and the supportive cellular microenvironment. In general, CD44 shows a membranous staining pattern in the UM-SCC-11A cells (Alexia 488, green fluorescence). The supportive cells (fibrocytes, HMVECs and HUVECs) were labeled using CD105 [HMVECs in (A) and HUVECs in (B); Cy 3, red fluorescence]. The nuclei were stained with DAPI. The cell-cell interactions that were observed were (A) broadly based cell contacts and (B) digitiform podia formations of UM-SCC-11A towards the supportive stromal cells. CSCs, cancer stem cells; HNSCC, head and neck squamous cell carcinoma; HMVEC, human microvascular vein endothelial cell; HUVEC, human umbilical vein endothelial cell.

The chemotaxis of the CD44^+^ UM-SCC-11A cells towards the SDF-1-expressing stromal cells was analyzed using a transwell migration assay. The CD44^+^ HNSCC UM-SCC-11A cell line was used as a model for CSCs, and their migration towards potentially supportive microenvironmental cells was evaluated. All the cell types that were tested secreted various concentrations of SDF-1 into the culture medium (m_fibro_, 1243.3±156.2 pg/ml; m_HMVEC_, 1061.4±23.2 pg/ml and m_HUVEC_, 849.6±110.9 pg/ml; Fig. 1). According to this, the migration of the UM-SCC-11A cells towards the supportive cells was increased by a higher supply of SDF-1 (contr_fibro_, 315.23±61.55 μm; m_fibro_, 477.73±143.7 μm; P_fibro_=0.003; contr_HMVEC_, 123.41±66,68 μm; m_HMVEC_, 249.04±111,95 μm; P_HMVEC_=0.004; contr_HUVEC_, 189.7±93.26 μm; m_HUVEC_, 260.82±161.58 μm; Fig. 2). A significantly larger amount of UM-SCC-11A cells migrated was towards the differentiated fibrocytes compared with the HMVECs or HUVECs (P_fibro/HMVEC_= 2.12E-11; P_fibro/HUVEC_=2.28E-5; Fig. 2).

## Discussion

The stem cell theory has become increasingly significant in tumor biology, particularly with regard to tumor development, progression and metastasis (18). Therefore, the CSC theory has provided new ideas and considerations for research and therapeutic options for malignant diseases (19). Currently, the presence of CSCs may not only be identified in hematological malignancies, but also in solid tumor entities (4,20). The CSC theory in solid tumors, such as HNSCC, has dramatic consequences. To date, therapeutic interventions, including surgery or chemoradiation, have been directed towards the bulk of the tumor without focusing on the small amount of specialized tumor cells, which have the facilities of self-renewal, differentiation and unlimited proliferation. However, the correct combination of markers that should be used to isolate the CSC in HNSCC remains unclear. The combination of markers appears to be different for different types of tumors (4,20,21). Furthermore, the presence of CSCs in HNSCC is postulated. Prince *et al* revealed that CD44^+^ cells, compared with CD44^−^ cells, were able to engraft a new HNSCC tumor in the mouse model (4). According to results of the study by Prince *et al*, CD44^+^ was sufficient to isolate cells with CSC properties out of the bulk of a HNSCC tumor. Following updated research, ALDH1 is another marker that has been postulated as a CSC marker in HNSCC (22). In the present study, CD44 was used as a CSC marker. In a previous study, a high expression of CD44, particularly at the invasive front of the tumor, was identified in HNSCC tissue samples (23), where the tumor cells were in contact with their surrounding cells, including the stromal and endothelial cells (24). The localization of CSC candidates at the border of the tumor is feasible, as this is where invasion and tumor growth occurs. Invasiveness and metastasis of a tumor also depends on the capacity to cut and rebuild the extracellular matrix (25,26). Malignant cells infiltrate healthy tissue by degrading components of the extracellular matrix, breaking down vessel borders and therefore generating metastases in distant organs. A number of tumor types have been shown to involve the presence of matrix metalloproteinases, including HNSCC (24,26,27).

The SDF-1-CXCR4 axis is involved in several aspects of tumor progression, including angiogenesis, metastasis and survival (28). The microenvironment of the bone marrow has been considered to support the survival, differentiation and proliferation of hematopoietic progenitor cells (29), as well as malignant progenitor cells of the hematopoietic system, including those of B-cell acute lymphoblastic leukemia (30). The pathway that includes the SDF-1-CXCR4 axis is postulated to be responsible for the retention of lymphoid and myeloid leukemia cells in the bone marrow (30,31). The significance of the SDF-1-CXCR4 axis is well-discussed in the hematopoietic system. However, to the best of our knowledge, the present study was the first to show that HNSCC cells also demonstrate podia formation as a pre-condition for locomotion using dose-dependent migration towards an SDF-1 gradient (14). In summary, the SDF-1-CXCR4 axis may also play a crucial role in the development, progress, invasion and metastasis of HNSCC and may be an essential pathway in the interaction between CSCs in HNSCC and the surrounding supportive niche.

In previous studies, CXCR4 was identified in the tumor nests of HNSCC, but not in the surrounding stroma of the CSC niche (23). Clatot *et al* revealed that the intratumoral level of SDF-1 correlated with survival in HNSCC (32). By contrast, the concentration of SDF-1 in the peripheral blood of HNSCC patients was not observed to differ in comparison with healthy donors (14). The latter findings are consistent with the results of the present study, which suggest that the SDF-1-CXCR4 axis may be involved in the CSC niche within the tumor, but not in the periphery of the blood system. In previous studies, and in the present *in vitro* model of the stem cell niche, we have shown that polarization and the formation of filopodia may be increased in the CD44^+^ CXCR4^+^ HNSCC UM-SCC-11A cell line in a dose-dependent manner using SDF-1 (10,14). This effect may be attributed to the cytoskeleton rearrangements of actin-containing protrusions (10,33) and may be influenced by extracellular factors, including matrix metalloproteinases (33,34). In general, podia formation is believed to interact with cell adhesion to the microenvironment (10). Evidence that podia formation and adhesion to the CSC niche plays a role in HNSCC CD44^+^ cells may improve the understanding of these interactions and offer insight into new strategies for cancer-directed therapy in HNSCC using small molecule agonists or antagonists of SDF-1 (10). These may be used to interfere with the CSC niche and cause the inhibition or prevention of tumor invasion and metastasis. Further experiments are required to expand and specify the cell-cell interactions in the CSC niche of solid tumors. It may be possible to develop strategies of therapy that are aimed at CSCs or the SDF-1-CXCR4 axis.
